# Epidemiological and Clinical Features of SARS-CoV-2: A Retrospective Study from East Karachi, Pakistan

**DOI:** 10.7759/cureus.8679

**Published:** 2020-06-17

**Authors:** Shumaila Tahir, Syeda Anjala Tahir, Taha Bin Arif, Bushra Majid, Zainab Majid, Farheen Malik, Ashfaque Ahmed, Arslan Memon, Jawad Ahmed

**Affiliations:** 1 Internal Medicine, District Health Office, East Karachi, PAK; 2 Internal Medicine, Civil Hospital Karachi, Karachi, PAK; 3 Internal Medicine, Dow University of Health Sciences, Karachi, PAK; 4 Preventive Medicine, Epidemiology and Public Health, District Health Office, East Karachi, PAK; 5 Public Health, District Health Office, East Karachi, PAK; 6 Public Health, COVID Control Room, East Karachi, PAK

**Keywords:** sars-cov-2 (severe acute respiratory syndrome coronavirus -2), covid-19, pakistan, clinical features, epidemiology, infection spread, isolation and quarantine, coronavirus disease, retrospective, clinical symptoms

## Abstract

Background

Severe acute respiratory syndrome coronavirus 2 (SARS-CoV-2) has spread to almost every country on the globe, and each country is reporting the symptomatic presentation of their patients to give better insight into the various clinical presentations of SARS-CoV-2. However, the epidemiological literature from Pakistan is scanty.

Methods

We retrospectively analyzed data from 412 patients who were residents of East Karachi and tested positive for SARS-CoV-2 between February 26 to April 24, 2020. Patients' demographics, symptoms, travel and contact history, and outcomes were recorded. All statistical analysis was performed using the Statistical Package for the Social Sciences (SPSS) version 22 (IBM SPSS Statistics for Windows, IBM Corp, Armonk, NY).

Results

Most of the patients were male (64.6%), the majority (43.3%) belonging to the 21- to 40-year age group. Most of the patients (65.5%) were residents of Gulshan Iqbal. A total of 15.8% of the patients were admitted to the hospital, and 3.9% of patients expired. The three most common presenting symptoms were fever (74.8%), cough (60.4%), and flu (35.5%). The majority of patients (89.3%) gave a history of contact with SARS-CoV-2 patients.

Conclusion

The number of SARS-CoV-2 cases is rapidly increasing in Karachi, Pakistan. There is a need to educate the population about the most common sign and symptoms of the virus so that individuals can identify these symptoms and get themselves tested. The concerned authorities should devise an adequate and effective plan to flatten the infectivity curve.

## Introduction

In December 2019, several locals in Wuhan, China presented to the hospital with respiratory symptoms due to a novel pathogen. The pathogen was identified and named as severe acute respiratory syndrome coronavirus 2 (SARS-CoV-2). SARS-CoV-2 is classified as a type of RNA virus, a member of the coronavirus family, and belongs to the "beta" genus. Other members of the same genus include SARS-CoV-1 and Middle East respiratory syndrome coronavirus (MERS-CoV) [1-3]. The possible origin of SARS-CoV-2 from bats could be elucidated by its similarity (88% identical) to two other SARS-like CoVs derived from bats (bat-SL-CoVZC45 and bat-SL-CoVZXC21) [1,2].

Since its origin, the virus has spread all over the world, affecting nearly every continent and triggering an international public health emergency in its wake. On March 11, the World Health Organization (WHO) declared coronavirus disease of 2019 (COVID-19) a pandemic. According to the most recent WHO COVID-19 situation reports (dated May 19, 2020), a total of 4,731,458 individuals have been infected, with 316,169 confirmed deaths [4]. In the Eastern Mediterranean region, Pakistan has the third-highest number of cases after Iran (122,492 cases) and Saudia Arabia (59,854 cases). Pakistan has 45,734 cases (17,947 in Sindh, 16,685 in Punjab, 6,554 in Khyber Pakhtunkhwa, 2,885 in Baluchistan, 689 in Gilgit-Baltistan and Azad Kashmir) with 985 deaths as of May 20, 2020 [5].

The rampant nature of SARS-CoV-2 is due to its high infectivity. The virus can be transmitted from human to human through physical contact, respiratory droplets produced by coughing, or sneezing [6]. Recently, vertical transmission through the mother to the newborn has also been postulated [7].

SARS-CoV, MERS-CoV, and the recently identified SARS-CoV-2 all cause respiratory symptoms. The main clinical manifestations of SAR-CoV-2 range from mild asymptomatic disease to life-threatening complications. Initially, the patient presents with cough, fever, dyspnea, and fatigue. Other less common complaints may include diarrhea, headache, and the production of sputum. The disease can progress to cause pneumonia, leukopenia, and lymphopenia [8]. Severe complications may occur, such as acute respiratory distress syndrome (ARDS), RNAaemia, acute cardiac injury (ACI), secondary infection, heart failure (HF), and multiple organ failure requiring intensive care unit (ICU) admissions [3,8]. 

Since its spread in Pakistan, the government has taken drastic measures for control and prevention of the further spread of COVID-19, including quarantine of suspected individuals carrying the disease and improvement in diagnostic and treatment procedures. Nonetheless, the already scarce resources of the healthcare system of Pakistan have been stretched thin. The primary aim of this retrospective observational study was to report the epidemiological features and statistics of individuals infected with COVID-19 from February 26 to April 24 from East Karachi, Pakistan, and contribute towards an accurate collection of figures from the country. We also aimed to study the age groups, modes of transmission, and durations and details of symptoms, among other variables, to understand this wide-spreading disease better and work effectively towards prevention and suitable management plans.

## Materials and methods

We carried out a retrospective cross-sectional analysis of the infected population of East Karachi (consisting of two towns, Gulshan Iqbal and Jamshed). All records that were found to be positive for the COVID-19 virus were analyzed, including cases that presented to the hospitals with screening facilities along with cases traced by the Rapid Response Team of the district's COVID-19 control room for the 'at home' suspects, cases and contacts. The data was collected from the daily notifications given to the surveillance line list and updated by the district health office, control room, which included data from the first case, from February 26 to April 24, 2020.

The individuals of East Karachi were filtered via screening criteria for suspected cases, due to the high cost and lack of testing kits, which evaluated the epidemiological history and clinical manifestations. The first part of criteria comprised the following four points: (1) Travel history from a high-risk area within 14 days before the disease onset (2) History of contact with individuals with SARS-CoV-2 (positive NAT result) within 14 days before the disease onset (3) History of contact with individuals with fever or respiratory symptoms in the high-risk area within 14 days before the disease onset (4) Disease clustering (two or more cases of fever and/or respiratory symptoms at home/school/office etc.). The clinical manifestations as part of the criteria are as mentioned: (1) Fever and/or respiratory symptom (2) Significant findings on CT imaging (3) Levels of white blood cell count normal or decreased.

Nasopharyngeal swabs were obtained from individuals with high suspicion for the COVID-19, which were tested using polymerase chain reaction (PCR) to confirm the diagnosis. The suspected or confirmed cases were clinically classified as asymptomatic, mild, moderate, severe, and critical, according to the National Institute of Health, Pakistan guidelines and are defined below in Table 1 [9].

**Table 1:** Clinical classification of confirmed/suspected cases into asymptomatic, mild, moderate and severe categories RT-PCR (Reverse transcription-polymerase chain reaction); CXR (chest X-ray); CURB-65 score (confusion, uremia, respiratory rate, BP, age ≥ 65 years); qSOFA (quick sequential organ failure assessment) score; PaO_2 _(partial pressure of arterial oxygen); FiO_2_ (percentage of inspired oxygen); PaCO_2_ (Partial pressure of carbon dioxide in arterial blood); JVP (jugular venous pressure); BP (blood pressure) Data in the above table adapted from [9].

Classification	Definition
Asymptomatic	Nasopharyngeal RT-PCR positive for COVID-19 without any symptoms
Mild	Presence of symptoms consistent with COVID-19 such as fever, fatigue, cough (with or without sputum production), anorexia, malaise, muscle pain, sore throat, dyspnea, nasal congestion, or headache without any hemodynamic compromise, need for oxygen or chest x-ray findings
Moderate	Hypoxia (oxygen saturation ≤ 94%) or mild infiltrate on CXR
	Persistent high-grade fever for over three days
Severe	Shortness of breath with hypoxia with moderate to severe pneumonia without meeting the criteria for critical disease
Critical	Presence of any of the following with COVID-19:
	1. Respiratory rate >30 breaths/minute
	2. Severe respiratory distress (can't speak in sentences)
	3. Central cyanosis
	4. Confusion, agitation, restlessness
	5. CURB-65 (3 or 4 score)
	6. qSOFA score of 2 or more
	7. Widespread infiltrates on CXR
	8. PaO_2_/FiO_2_ ratio less than 300, or PaO_2 _less than 65 or Rising PaCo_2_
	9. Evidence of heart failure (Raised JVP, Gallop rhythm)
	10. Signs of shock: delayed capillary refill; cold, clammy peripheries; mottled skin; systolic BP less than 90 or less than 40 mmHg of baseline in hypertensive; urine output < 0.5 ml/kg/hr

Due to the lack of resources, including the availability of health workers, personal protective equipment (PPE), and adequate isolation spaces in hospitals, it was proposed to manage asymptomatic and mild cases via home isolation. In contrast, moderate, severe, and critical cases were admitted to a hospital facility. A patient was labeled as recovered upon testing negative for COVID-19 on two separate samples after contracting the disease. Patients who tested positive for the infection and succumbed to the disease were classified as "expired", contributing to the mortality rate. The criteria mentioned above and guidelines were evolved and developed over time, the main framework being consistent with all cases.

All statistical analysis was performed using the Statistical Package for the Social Sciences (SPSS) version 22 (IBM SPSS Statistics for Windows, IBM Corp, Armonk, NY). For the assessment of qualitative variables, frequencies and percentages were used.

## Results

We analyzed the data of 412 COVID-19 patients in our study. The majority of the study participants were male (n=266, 64.6%). Additionally, the majority of participants were also young (21 to 40 years [n=179, 43.5%]; 41 to 60 years [n=98, 23.8%]). All participants came from two towns in East Karachi, with more than half belonging to Gulshan Iqbal (n=270, 65.5%), as represented in Table 2.

**Table 2:** Demographics of participants (n=412)

Variables	Options	No of participants (n)	Percentage (%)
Gender	Male	266	64.6
	Female	146	35.4
Age (years)	0-20	64	15.5
	21-40	179	43.5
	41-60	98	23.8
	Greater than 60	71	17.2
Town	Gulshan Iqbal	270	65.5
	Jamshed	142	34.5

Number of cases reported

**Figure 1:**
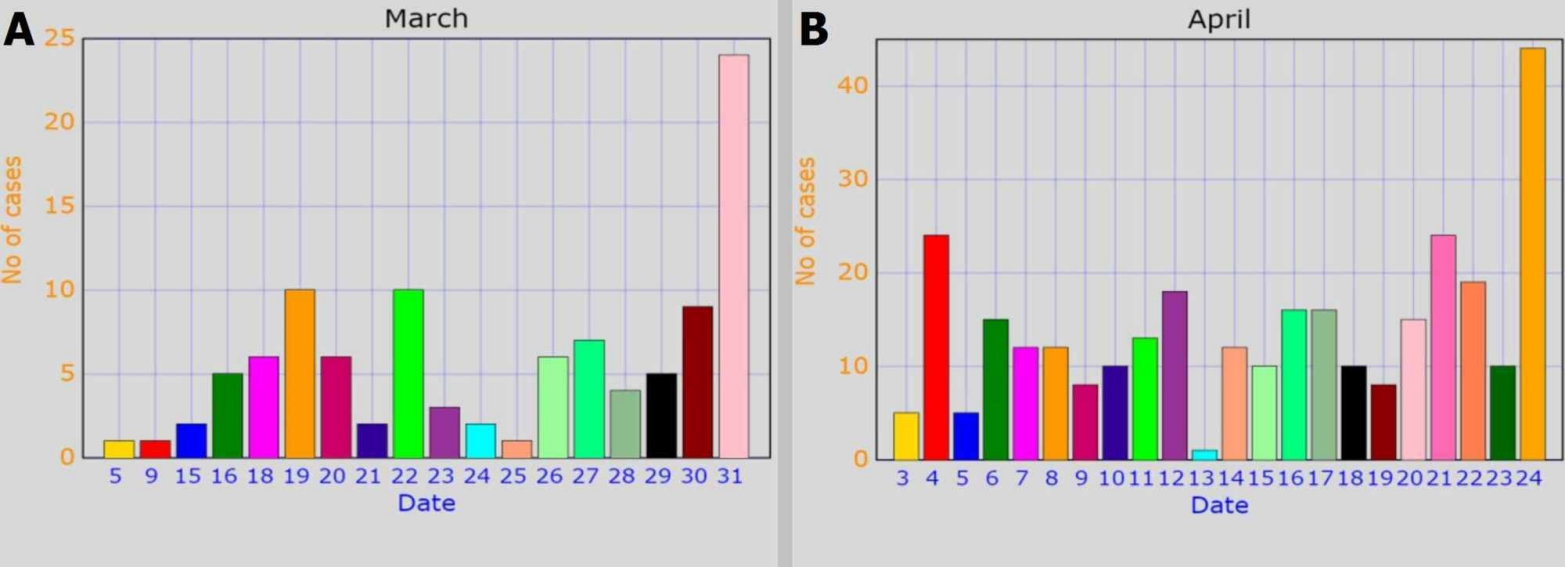
Number of COVID-19 cases reported in (A) March and (B) April

Data collection was started from February 26, 2020, and one case was reported that day. During the month of March, 104 cases were reported, while 307 cases were reported till April 24, 2020, as is illustrated by Figure 1.

Status of participants

The differing status of our study participants, all of whom were positive for COVID-19, were categorized into four groups. The groups included participants who had recovered, expired, sent into isolation at home, and admitted to the hospital, as shown in Table 3.

**Table 3:** Status of COVID-19 patients

Status	No of patients (n)	Percentage (%)
Recovered	92	22.3
Expired	16	3.9
Home isolation	239	58.0
Admitted in hospital	65	15.8
Total	412	100

Symptoms of patients

Most of the patients presented with at least one symptom (n=375, 91.0%), whereas only 9.0% (n=37) were asymptomatic. These symptoms varied and the most common that were experienced were fever (n=308, 74.8%), cough (n=249, 60.4%), flu (n=138, 33.5%), and body ache (n=109, 26.5%). The least common symptoms witnessed were nausea (n=8, 1.9%), headache (n=5, 1.2%), chest pain (n=4, 1.0%), decreased appetite (n=2, 0.5%), and lower respiratory tract infection (LRTI) (n=2, 0.5%). The symptoms are listed below in Table 4.

**Table 4:** Symptoms of COVID-19 patients

Symptoms	Frequency (%)		Symptoms	Frequency (%)	
	Yes	No		Yes	No
Cough	249 (60.4)	163 (39.6)	Drowsiness	9 (2.2)	403 (97.8)
Fever	308 (74.8)	104 (25.2)	Sore throat	9 (2.2)	403 (97.8)
Bodyache	109 (26.5)	303 (73.5)	Shortness of breath	53 (12.9)	359 (87.1)
Flu	138 (33.5)	274 (66.5)	Diarrhoea	27 (6.6)	385 (93.4)
Vomit	20 (4.9)	392 (95.1)	Lower respiratory tract infection	2 (0.5)	410 (99.5)
Abdominal pain	17 (4.1)	395 (95.9)	Headache	5 (1.2)	407 (98.8)
Fatigue	24 (5.8)	388 (94.2)	Decreased appetite	2 (0.5)	410 (99.5)
Arthralgia	41 (10.0)	371 (90.0)	Chest pain	4 (1.0)	408 (99.0)
Nausea	8 (1.9)	404 (98.1)	Chest congestion	55 (13.3)	357 (86.7)
Upper respiratory tract infection	9 (2.2)	403 (97.8)	-	-	-

History of travel/contact

The vast majority of the patients (n=368, 89.3%) had been exposed to the virus from a person who was COVID-19 positive. Others had a positive travel history or had attended a big religious congregation (Table 5).

**Table 5:** Travel/contact history of COVID-19 patients

History		Frequency (n)	Percentage (%)
Contact with COVID-positive person		368	89.3
Travel history	Saudi Arabia	6	1.5
	Iran	5	1.2
	United States of America	5	1.2
	Australia	2	0.5
	United Kingdom	5	1.2
	Turkey	5	1.2
	Dubai	1	0.2
	Local travel	1	0.2
Religious congregation		14	3.4

## Discussion

The COVID-19 pandemic was confirmed to have reached Pakistan on February 26, 2020. As of May 19, there have been 43,966 cases with 1,841 recoveries and 939 deaths in the country [4]. With a rapid increase in the number of cases and a fragile economy, the country was put under a nationwide lockdown on April 1, 2020. Although Pakistan was expecting 50,000 cases of COVID-19 by April 25, the number remained less than half of the presumption [10]. The government of Pakistan has been taking necessary measures to control the outbreak and facilitate its people. Many hospitals across the country are working to combat the deadly outbreak of COVID-19. Early case detection, contact tracing, risk communication, social distancing, isolation and quarantine, and introduction of COVID-19 relief funds to receive donations for the welfare of people are other significant measures taken by the country [11].

Our study primarily highlights the scenario of COVID-19 cases in the East district of Karachi, Pakistan. On analyzing the demographic profile of patients, males were found to be affected twice as much (64.6%) as females (35.4%). This is significantly higher than a study from Wuhan, China, which indicated that 56% of patients with COVID-19 were males [12]. Similarly, another study of 140 patients from Wuhan found that 50.7% were males [13]. Gender is a significant risk factor of severity and mortality in patients with COVID-19. According to a retrospective study by Jin et al., men tended to develop more severe disease. Although men and women had similar susceptibility, men were more prone to dying and accounted for approximately 2.4 times that of women in the deceased patients [14]. COVID-19 can lead to hospitalization and even death in young and middle-aged adults. It has caused the most severe health issues for adults over the age of 60, with higher fatality over the age of 80. Comorbidities like diabetes, hypertension, heart diseases, or other chronic illnesses can cause more intense manifestations and complications in the disease. Besides, older adults are more susceptible to infection due to a gradual loss of resilience of the immune system. Almost a quarter (23.3%) of Italy's population is over 65 years where a significantly higher number of COVID-19 cases and deaths were reported with case fatality rate (CFR) of 0.7% in 40-49 years, 27.7% in >80 years, and 96.9% deaths occurring in adults aged 60 years and greater [15]. In contrast, approximately 38.04% population of Pakistan falls in 25-54 years age group. Our study reported a higher incidence of infection in 21-40 years depicting that age structure is a significant risk factor for incidence and mortality rates of COVID-19.

According to the WHO COVID-19 situation report, a total of 750,890 confirmed cases and 36,405 deaths were reported across the globe till March 31, 2020. These figures are approximately nine times greater than the statistics recorded on February 29, 2020. By the end of March, Pakistan had 2,039 confirmed cases with 82 recoveries and 26 deaths [4]. About 104 cases were reported in March from the eastern towns of Karachi, as demonstrated by our study. The global cases increased dramatically to around 2,626,321 confirmed cases by April 24, 2020. Pakistan crossed a critical mark of the outbreak in the country as the total number of cases surged to about 11,155 on April 24 with an immediate increase in death rate (237 deaths) secondary to COVID-19 [4]. However, our study recorded an approximately three-fold increase in the cases in East Karachi with the death of 16 individuals. Out of 412 cases, more than half (58%) were sent to home isolation, and 22% recovered while 15.8% were admitted to the hospital for supportive care. The rising COVID-19 cases pose a challenge to Pakistan's crumbling healthcare system. Fragile economy, lack of availability of personal protective equipment for healthcare providers, inadequate quarantine and testing facilities, and periodic ease in lockdown are the principal reasons for the drastic rise in cases [11].

Clinical presentations of COVID-19 range from asymptomatic or mild symptoms to complicated illness and/or mortality. Common symptoms include fever, cough, and shortness of breath, malaise, and respiratory distress. According to the Centers for Disease Control and Prevention (CDC), cough and shortness of breath with at least two accessory symptoms (fever, chills, sore throat, muscle pain, headache, and new loss of taste or smell) may indicate COVID-19. It can also present as gastrointestinal complaints like nausea, vomiting, anorexia, and diarrhea [16]. Symptoms may develop two days to two weeks following exposure to the virus. A pooled analysis of COVID-19 cases from January 4 to February 24, 2020, reported a mean incubation of 5.1 days. Out of 181 confirmed cases, 97.5% of individuals developed symptoms within 11.5 days [17]. The majority of patients in our study presented with a single complaint. Fever (74.8%) was the predominant symptom, followed by cough (60.4%), flu (33.5%), and body ache (26.5%). These findings coincide with an initial report by Huang et al., which specified fever (98%) as the most common clinical finding, followed by cough (76%) and myalgia (44%) [8]. Asymptomatic infections have been reported, for example, by Chan et al., but the exact incidence is unknown [18]. Our study found 37 individuals with no symptoms of COVID-19. Some unusual symptoms included abdominal pain, vomiting, arthralgia, drowsiness, diarrhea, headache, and anorexia.

Clinicians evaluating patients with fever and acute respiratory disease should obtain details regarding travel history or exposure to an individual who recently returned from a country experiencing active local transmission [19]. Furthermore, the CDC has proposed contact tracing as a part of a multipronged approach to fighting the COVID-19 pandemic [20]. Patients with suspected COVID-19 should be immediately reported to the healthcare provider and the local or state health department. Candidates with fever, symptoms of lower respiratory illness, and a travel history to Wuhan, China or other countries with uncontrolled COVID-19 cases or who have been in contact with an individual suspected of COVID-19 or with laboratory-confirmed COVID-19 in the preceding 14 days should be isolated and tested for the infection promptly [19]. One of the preventive measures proposed by WHO for protection from the spread of COVID-19 is the maintenance of an adequate distance of 1 m (3 feet) from others and avoiding crowded places [21]. About 89.3% of patients in our study contracted the disease from individuals with an existing infection, with a minority of patients reporting a history of travel to COVID-19 endemic states or attending religious congregations. The easing of the lockdown across Pakistan resulted in 1,637 new cases and 24 new deaths in one day; experts say if social distancing and isolation are not practiced by the overwhelming majority of the population, Pakistan would likely experience massive fatalities soon [22].

Our study has a few limitations. First, it includes all COVID-19 cases from only one district (i.e., East Karachi), Pakistan. A survey with a large sample size from multiple towns of the city can reflect more accurate statistical figures regarding the epidemiological and clinical features of COVID-19. Furthermore, the duration of the study was only two months. Detailed analysis of the clinical features, laboratory findings, complications, and prognostic indicators is recommended to get a better insight into the infection and its associated entities, which can help the state to think of possible strategies to combat the pandemic. A previous study from Karachi has depicted poor knowledge regarding the maintenance of adequate hygiene among healthcare providers [23]. Apart from maintaining social distancing, it is recommended to practice all necessary preventive measures like frequent cleansing of hands with running water or a sanitizer, covering coughs and sneezes with disposable tissues or clothing, and avoiding excessive touching of eyes and nose or unprotected contact with animals.

## Conclusions

SARS-CoV-2 is infecting the population of Karachi rapidly, resulting in an exponential increase in the number of cases. The descriptive analysis of the epidemiological data in our study showed a large majority contracting the virus through contact with an already infected person and highlights the significance of following the guidelines of maintaining social distancing. The public, government, and health authorities of Pakistan urgently need to realize their responsibilities and devise efficient plans to curb further spread of the disease, keeping in mind the limited resources and grave health outcomes.
